# Washed up: the end of an era for adrenal incidentaloma CT

**DOI:** 10.1186/s13244-025-02015-4

**Published:** 2025-06-27

**Authors:** James H. Seow, Damien L. Stella, Christopher J. Welman, Arjuna J. Somasundaram, Jan F. Gerstenmaier

**Affiliations:** 1 Department of Radiology, Royal Perth Hospital, Perth, Australia; 2 Department of Radiology, University of Melbourne, Melbourne, Australia; 3 Department of Radiology, Royal Melbourne Hospital, Melbourne, Australia; 4 Department of Medical Imaging, Fiona Stanley Fremantle Hospitals Group, Murdoch, Australia; 5 Department of Medical Imaging, Royal Brisbane and Women’s Hospital, Brisbane, Australia; 6 Griffith University, Gold Coast, Australia; 7 Department of Radiology, Alfred Health, Melbourne, Australia

**Keywords:** Adrenal incidentaloma, Adrenal gland neoplasms, Tomography (x-ray computed)

## Abstract

**Abstract:**

For over 20 years, the two key tenets of adrenal incidentaloma (AI) evaluation have been the upper threshold of 10 Hounsfield units (HU) on noncontrast CT (ncCT) to delineate benignity, and the utilisation of adrenal washout CT (AWCT) to evaluate those above this cutoff. In light of growing recent evidence that challenges these two traditional principles, as well as re-evaluation of the data that led to their acceptance, we conclude that neither of these mainstays of adrenal CT remains relevant in modern AI diagnostic workup. With an appropriate definition of an incidentaloma and endocrine assessment for the majority of adrenal lesions, our analysis establishes that the use of AWCT should be ceased in the assessment of AIs, and that a 20 HU attenuation threshold for lesions < 4 cm should replace the traditional 10 HU threshold to exclude malignancy in this patient population. We therefore propose new recommendations for the management of AIs based primarily on CT attenuation and lesion size on ncCT.

**Critical relevance statement:**

Increasing the CT attenuation threshold to 20 HU for lesions < 4 cm and eliminating washout CT for true adrenal incidentalomas, together with recommendations for endocrine assessment, will significantly decrease the over-investigation of overwhelmingly benign adrenal lesions, whilst confidently excluding malignancy.

**Key Points:**

- True incidentalomas exclude current or prior extra-adrenal malignancy and clinically suspected adrenal disease.
- Adrenal washout CT was never proven in the malignancy-sparse true incidentaloma population.
- Hormonal correlation in parallel with < 20 HU and < 4  cm thresholds of homogeneous lesions on noncontrast CT excludes malignancy.

**Graphical Abstract:**

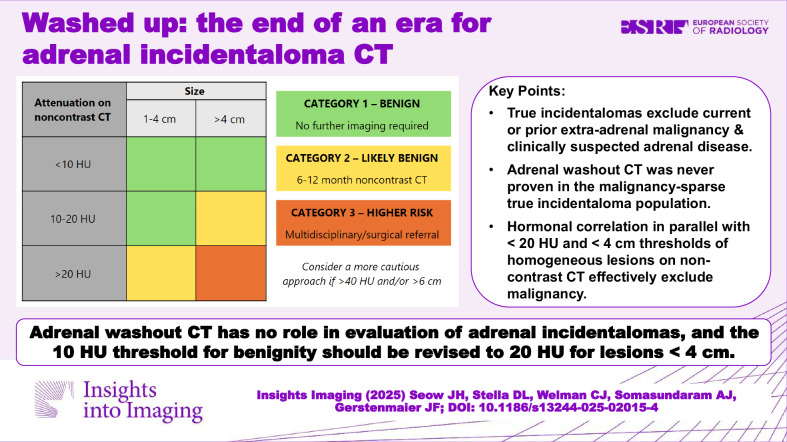

## Introduction

A 2002 paper encapsulated the prevailing views at the time regarding the two key principles of adrenal CT: a 10 Hounsfield unit (HU) upper threshold for lipid-rich adenomas (LRA), and adrenal washout calculation for the remainder. The authors stated, “with a combination of unenhanced and delayed enhanced CT, nearly all adrenal masses can be correctly categorised as adenomas or nonadenomas” [1].

Over 20 years later, this practice remains unchanged in many institutions and international guidelines [2–5]. However, on review of the interval published evidence and reanalysis of the foundational literature, we believe it is time to re-evaluate these tenets of adrenal incidentaloma (AI) imaging, proposing that the use of adrenal washout CT (AWCT) be ceased, and that the 10 HU threshold be revised upward.

In this paper, we revisit the history, established principles and international guidelines pertaining to adrenal imaging with a focus on CT and reinforce the necessity of endocrine assessment for the majority of AIs. We subsequently challenge the evidence behind AWCT, demonstrating its inherent inaccuracy, and highlight that the technique was never proven in the malignancy-sparse true AI population. We then provide substantiation of 20 HU as a new threshold for benign AIs measuring < 4 cm.

## Methodology

This narrative review specifically targeted the evidence behind AWCT and attenuation thresholds on noncontrast CT (ncCT) by identifying the seminal papers that described their establishment and evaluating the cited literature within the major historic and current AI international guidelines (J.S., D.S.). Additional literature was retrieved with library assistance (South & East Metropolitan Health Service Library and Information Service) using Medline, PubMed, Web of Science, Trip Medical Database, Embase and cross-correlation with Google Scholar databases from 2023 to September 2024. Search criteria included “adrenal”, “nodule”, “lesion”, “adenoma”, “lipid poor”, “lipid rich”, “nonadenoma”, “wash-in”, “washout”, “phaeochromocytoma”, “carcinoma”, “adrenal incidentaloma*” (J.S., C.W.). The search was constrained to title, abstract, keyword, key field, and English. Acceptance for inclusion was based on consensus discussion between all authors.

## Definition of adrenal incidentalomas

Adrenal lesions (AL) are identified in up to 4–5% of CT examinations [6–8] and encountered in three main clinical scenarios. First, ALs may result in clinically evident symptoms, primarily endocrine in nature. Second, ALs may be identified in patients with extra-adrenal malignancy, although unless there are also other metastases, most ALs are still benign in this setting [9].

However, third and most frequently, ALs are incidental findings, though what constitutes an incidentaloma varies markedly in the literature. For example, a definition of “an adrenal mass detected on imaging not performed for a suspected adrenal disease” [3] excludes suspected functional lesions, but without further qualifiers, classifies unsuspected adrenal metastases in those with known malignancy as incidentalomas.

Alternatively, the Society of Abdominal Radiology’s AI definition is “an incidentally detected adrenal nodule or mass that is unrelated to the clinical indication for the imaging examination performed”, thereby excluding adrenal metastases detected in cancer staging [10]. However, imaging performed to confirm diverticulitis in someone with recent lung cancer bypasses this definition. Furthermore, both definitions may include those with no previously known malignancy, but with unequivocal extra-adrenal malignancy on current imaging.

We therefore propose that an optimal AI definition is one that excludes patients with current or prior extra-adrenal malignancy or clinically suspected adrenal disease. Additionally, we conform to published standards that AIs measure ≥ 1 cm [2, 3, 10], with a recent study finding this to be a clinically appropriate threshold [11].

## The history of adrenal imaging

Adrenal imaging began over a century ago with air-insufflation radiography in the 1920s [12] and then incorporated tomography in the 1940s [13]. CT provided the first published cross-sectional image of an adrenal gland in 1976 [14], and the first specific lesion in 1977—a macroscopic fat-containing myelolipoma [15].

Reports of hypoattenuating ALs (without macroscopic fat) followed, with recognition they correlated with benign adrenocortical adenomas [16, 17]. By 1991, a 10 HU cutoff had been proposed for differentiating benign and malignant lesions [18], and whilst alternative levels were suggested [19], the 10 HU threshold became widely accepted in international radiological and clinical guidelines [2, 4, 5, 20–23].

Dynamic contrast-enhanced evaluation of ALs began with MRI in the early 1990s [24–26], whilst the development of AWCT appeared less intentional. Routine post-contrast lesion attenuation was deemed unhelpful, therefore requiring performance of ncCT on separate days [27]. Publications subsequently evaluated the utility of lesion attenuation assessment after diminishing 60-, 30-, and 12–18-min delays [28–30].

Key papers by Korobkin et al and Szolar et al in 1998 then firmly established the AWCT technique, leading Caoili et al to conclude that nearly all adenomas and non-adenomas could be differentiated [1, 31, 32]. Whilst there was some technique variation, the dominating protocol involved portal venous (PV) and 15-min phases, and with Absolute Percentage Washout (APW) > 60% and Relative Percentage Washout (RPW) > 40% considered indicative of adenomas [10, 21].

These principles of adrenal imaging: a 10 HU threshold and AWCT, have essentially remained unchanged over the last two decades, as reinforced by current international guidelines [2–5].

## Adrenal incidentaloma guidelines and endocrine evaluation

Initial AI guidelines were conservative, with the combined American Associations of Clinical Endocrinologists and Endocrine Surgeons 2009 publication recommending re-imaging of radiologically benign lesions (< 4 cm and < 10 HU), at 3–6 months, then annually for 1–2 years [20], a stance that has perpetuated as recently as 2017 [33].

The American College of Radiology 2010 Incidental Findings White Paper and 2017 update, however, proposed that radiologically benign lesions: myelolipomas, non-enhancing lesions ± calcification, those ≤ 10 HU (or with MRI opposed-phase signal loss), and lesions with ≥ 1 year stability did not require imaging follow-up. Notably, they also recommended AWCT for lesions > 10 HU [2, 21].

Similarly, AWCT and the 10 HU threshold were incorporated into the 2011 & 2023 Canadian Urological Association, 2016 & 2023 European Society of Endocrinology (ESE)/European Network for the Study of Adrenal Tumors (ENSAT), 2017 Korean Endocrinology Society and 2023 BMJ Best Practice guidelines [3–5, 22, 23, 34, 35].

Additionally, these guidelines all recommend initial hormone testing for AIs, as approximately 15% are hormonally active [36, 37], and 20–50% of adenomas may cause mild autonomous cortisol secretion (MACS) [3]. Whilst the details lie beyond this article’s scope, the recommended initial endocrine assessment includes: clinical examination for hormone excess; testing for cortisol secretion and metanephrines; and aldosterone/renin ratios, sex-hormones and steroid precursors in select populations (Table S1) [3]. In the 2023 ESE revision [3], metanephrine assessment for lesions < 10 HU was removed, following studies demonstrating phaeochromocytomas (PCCs) were consistently above this threshold [38–44].

We therefore reiterate that radiology reports for all AIs (excluding non-adenomatous benign aetiologies, such as myelolipomas and cysts) include a generalised recommendation for hormonal evaluation. In one study, this improved rates of endocrine testing from 13.9 to 48.0%, whilst 100% were evaluated if seen by endocrinologists, implying additional benefits of recommending endocrinologist referral [45].

## The problem with phaeochromocytomas

The first challenge to the tenets of adrenal imaging arose with reports of washout in PCCs mimicking adenomas, culminating in a 2018 meta-analysis evaluating 10 studies, 1017 patients and 114 PCCs [46]. Of the studies with “strict” AWCT technique, 47% of PCCs met adenoma washout criteria. A 2019 study of pathologically proven PCCs similarly found that 50% would be misdiagnosed on AWCT overall, and 80% of those < 3 cm [47]. A further international ENSAT study, although reporting a lower 28.9% rate, still concluded that AWCT was unreliable for excluding PCCs [48].

Consequently, additional CT criteria for PCCs have been evaluated, including: noncontrast, arterial, PV and delayed attenuation thresholds, and enhancement ratios [40, 49–56]. However, several criteria significantly traded off specificity for sensitivity, others reported conflicting results, some relied on AWCT for diagnosis, whilst others artificially excluded alternative pathologies. A revealing finding in one study was that although size and noncontrast attenuation thresholds had limited performance in differentiating PCC from lipid-poor adenomas (LPA) (AUC 0.781 and 0.845, respectively), they both outperformed all enhancement criteria (AUC range 0.600–0.727), therefore questioning any utility of contrast assessment [54].

MRI diagnosis is also unreliable, as 35% of PCC may lack classical high T2-signal [57], and in a recent small study, > 50% had ‘atypical’ MRI appearances [58]. A proposed quantitative T2-signal and entropy calculator has also had overall mixed results [59–62].

Despite these challenges of differentiating between PCCs and adenomas with CT/MRI, importantly, only 10–20% of PCCs are clinically silent, and urinary and plasma metanephrine testing have reported 97–99% sensitivity [63, 64]. Given that all AIs > 10 HU require metanephrine testing, the real-world consequences are therefore limited.

## Is adrenal washout CT washed up?

Beyond PCC, AWCT has even poorer diagnostic performance with other classically hypervascular lesions, such as renal-cell and hepatocellular carcinoma metastases, with up to 95% meeting adenoma APW criteria [65]. Further confounding their differentiation is that these may also contain metastatic lipid, which does not necessarily correlate with primary tumour adiposity [66].

However, even when including non-hypervascular lesions, in a study evaluating 142 mixed tumours > 10 HU, 43% of benign lesions did not wash out, and 22% of malignancies did wash out, resulting in AWCT misclassifying 36% of all masses [67]. They concluded, AWCT “with the established thresholds for APW and RPW is insufficient to reliably diagnose adrenal masses”.

Additionally, studies have largely evaluated AWCT in artificially enriched populations, partly to achieve statistical significance. However, as ALs are most frequently encountered incidentally, a landmark paper by Corwin et al evaluated AWCT in a pure AI population [68]. In this multi-institution study, utilising reference standards of histopathology (*n* = 54), imaging follow-up > 1 year (*n* = 269; *x̄* = 4.6 years) or clinical follow-up > 5 years (*n* = 13; *x̄* = 8.0 years), 336 AIs > 10 HU and > 10 mm (*x̄* = 22 mm) were identified. The vast majority were benign (95.8%, *n* = 322), with 2.7% PCCs (*n* = 9), and only 1.5% malignant (3 adrenocortical carcinomas (ACCs), 3 metastases). Furthermore, in those < 4 cm, the malignancy prevalence was only 0.3% (*n* = 1), despite the inherently higher risk > 10 HU population.

Acknowledging the high sensitivity of metanephrines for PCC, the prevalence of non-PCC malignancy was not statistically different in nodules < 4 cm with APW > 60% (0%, *n* = 0) versus with APW < 60% (1.3%, *n* = 1). Additionally, even in masses > 4 cm with an inherently higher malignancy rate, there was no significant difference between those with (16.7%) and without washout (23.1%). Importantly, when excluding PCC, for nodules < 4 cm, the negative predictive value (NPV) of washout (for benignity) was only 1.4%, meaning that with a supposed “positive” malignancy result, 98.6% of lesions were still benign.

The utility of AWCT for AIs therefore appears negligible, due to both its inherent limited accuracy and the extremely low malignancy prevalence in true AI populations [68–70]. Notably, this low malignancy prevalence was established as early as 2008 by Song et al, who found no malignant lesions in 1049 consecutive true AIs [8]. Whilst these findings appear disparate to the seminal studies that established AWCT, re-evaluation of these reveals each had highly enriched proportions of malignancies, and furthermore were dominated by LRAs, for which AWCT is no longer recommended. In fact, disproportionately, each had more malignancies than LPAs (malignancy:LPA ratios in Szolar et al, Korobkin et al and Caoili et al, respectively 54:33, 22:7, 36:22) [1, 31, 32]. AWCT was therefore never proven in the true malignancy-sparse AI population.

Furthermore, this low malignancy rate is not unexpected, considering primary adrenal malignancy (ACC) is extremely rare (annual incidence 0.5–2.0 cases/million) [71, 72], but moreover up to 60% are hormonally active [71, 72], and in two studies, 81.9–89.4% were symptomatic (with median size 8.5–12 cm) [71, 73], and therefore few are clinically incidental. Additionally, secondary malignancy (metastases), although much more common, is extremely unlikely to present as isolated adrenal disease. Specifically, in 1715 patients with unknown primary cancers, whilst 5.8% had adrenal metastases, only 0.2% had isolated adrenal involvement, and these were all > 6 cm, mostly bilateral, and retrospectively all symptomatic [74]. Truly incidental isolated adrenal metastases therefore are essentially non-existent, and combined with the rarity of asymptomatic ACC, result in a minimal AI malignancy rate.

In light of these findings, we recommend that the use of AWCT in the true AI population be ceased.

## CT attenuation threshold: 20 is the new 10

With AWCT’s credibility doubtful, we question the second tenet of adrenal CT: the 10 HU threshold for LRAs and benignity in general. The 2023 ESE guidelines reported pooled sensitivities and specificities for a 20 HU threshold for malignancy of 96.8% and 76.7%, respectively, versus 100% and 57.5% for 10 HU, based on five key papers [3]. In addition, through expansion of the ESE search strategy to cover more recent studies, we identified one additional paper with data relevant to the 20 HU threshold [37]. These six papers (Tables 1, 2) are individually readdressed here for two reasons.

**Table 1:** Study population data for studies reporting 20 HU attenuation and 4 cm size thresholds for discrimination between benign and malignant adrenal lesions

Study (author [ref.])	Study population selection criteria	Non-incidental and functional subpopulations (if known)	Estimated true AI subpopulation	Histopathology reference standard (%)	PCC (%)	Non-PCC malignancy (%)
Marty et al [77]	233 patients with 253 “AIs” ≥ 1 cm referred to endocrinology (after exclusion of 184 patients without histopathology or adequate follow-up).	10.7% patients (*n* = 22) had a history of cancer considered in remission, including 1 prior contralateral ACC^a^ At least 27.5% patients (*n* = 64) had hormone production (62 requiring surgery/biopsy + 2 biochemically diagnosed PCC)^b^	Unknown—endocrinology referred, surgically enriched population	50.6% (*n* = 118; 115 surgery, 3 biopsy)	13.0% (*n* = 33/253)	11.1% (*n* = 28/253; 23 ACC, 1 mets, 1 bilateral lymphoma, 2 other)
Schloetelburg et al [67]	216 patients with 252 “adrenal lesions” ≥ 1 cm with washout CT (after exclusion of 48 patients without histopathology or adequate follow-up).	18.7% (*n* = 47) lesions in patients with current extra-adrenal malignancy^a^ 12.3% (*n* = 31) lesions in patients with suspected adrenal disease^a^	At most, 69.0%^a^	36.5% (*n* = 92)	4.4% (*n* = 11/252)	15.1% (*n* = 38/252; 9 ACC, 25 mets, 4 lymphoma)
Ebbehoj et al [75]	1287 patients with an “adrenal tumour” (any size) from population health records (660 with unenhanced CT).	12.1% (*n* = 156) detected in cancer staging or follow-up^a^ 4.8% (*n* = 62) in investigation of hormone excess symptoms^a^ 1.5% (*n* = 19) “other”, e.g., postmortem, in surgery, other symptoms^a^	At most, 81.6%^a^	43.2% (*n* = 48/111) of malignant lesions (as per supplementary appendix)^b^ Not stated for benign lesions	1.1% (*n* = 14/1287)	8.6% (*n* = 111/1287; 4 ACC, 96 mets, 4 lymphoma, 7 neuroblastoma) In “AI” subgroup, 3.3% (*n* = 35/1050; 1 ACC; 30 mets; 1 lymphoma, 3 neuroblastoma) In “cancer” subgroup, 42.9% (*n* = 67/156; 0 ACC, 62 mets, 3 lymphoma, 2 neuroblastoma)
Bancos et al [76]	2017 patients from specialist centres with a “newly identified adrenal mass” > 1 cm. Excluded patients with biochemical evidence of PCC, pregnancy, lactation, steroid affecting drugs. Excluded CTs for cancer staging/monitoring.	16.4% (*n* = 331) “non-incidental”—imaging for clinical signs and symptoms of steroid excess or a tumour^a^	At most, 83.6%^a^	29.0% (*n* = 585/2017)^b^	0.5% (*n* = 10/2017)	8.1% (*n* = 163/2017; 98 ACC, 39 mets, 8 lymphoma, 18 other)
Hong et al [78]	1149 patients with “newly diagnosed AIs”. Excluded suspected adrenal pathology and history of extra-adrenal malignancy.	30.5% functional (benign + ACC; *n* = (348 + 2)/1149)^b^ including 4.5% with overt Cushing with Cushingoid features (benign + ACC; *n* = (50 + 2)/1149) 11.5% had primary aldosteronism (*n* = 132) and 7.1% MACS (*n* = 82)^a^	At most 95.5%^b^	Not stated	7.3% (*n* = 84/1149)	1.7% (*n* = 20/1149; 14 ACC, 3 lymphoma, 2 leiomyosarcoma, 1 neuroblastoma, 0 mets)
Kahramangil et al [37]	2219 patients with “AIs” ≥ 1 cm. Excluded known active malignancy, signs and symptoms of hormonal hyperfunction, pain due to an adrenal mass.	15.7% functional (of those worked up); no overt Cushing	Possibly all	At least 25.9% (*n* = 574; 382 immediate and 192 delayed surgery)^b^	4.4% (*n* = 97/2219)	2.2% (*n* = 49/2219; 38 ACC, 9 mets, 2 lymphoma, as per supplementary table)

Studies ordered from lowest to highest estimated population of true AIs

*AI* adrenal incidentaloma, *ACC* adrenocortical carcinoma, *PCC* phaeochromocytoma, *MACS* mild autonomous cortisol secretion, *mets* metastases

^a^ Provided data

^b^ Calculated from data

**Table 2:** Threshold data for studies reporting 20 HU attenuation and 4 cm size thresholds for discrimination between benign and malignant adrenal lesions

Study (author [ref.])	Estimated true AI subpopulation	HU thresholds	Size thresholds	Combined thresholds
Marty et al [77]	Unknown—endocrinology referred, surgically enriched population	≤ 15 HU = 100% PPV for ‘adenoma’^a,c^ ≤ **20 HU** = **96.2% PPV for ‘adenoma’**^**a,c**^ (Note PCC included in ‘non-adenomas’)	≤ 3 cm = 93.4% PPV for ‘adenoma’^a,c^ ≤ **4** **cm** = **89.4% PPV for ‘adenoma’**^**a,c**^ (Note PCC included in ‘non-adenomas’)	≤ 3 cm and ≤ 20 HU = 100% PPV for ‘adenoma’^a,c^ ≤ 4 cm and ≤ 15 HU = 100% PPV for ‘adenoma’^a,c^ **≤ 4** **cm and** ≤ **20 HU** = **98.6% PPV for ‘adenoma’**^a,c^ (Note PCC included in ‘non-adenomas’)
Schloetelburg et al [67]	At most, 69.0%^a^	**≤ 20 HU** = **99.0% PPV for benignity**^**a**^ (Note PCC considered malignant: 1x PCC and 1x metastasis < 20 HU)	**< 4** **cm** = **87.9% PPV for benignity**^**a**^ (Note PCC considered malignant)	**None** (but ≤ 20 HU alone = 99.0% PPV for benignity^a^)
Ebbehoj et al [75]	At most, 81.6%^a^	**< 20 HU** = **99.4% PPV for benignity**^**b**^ (3 of 518 malignant, all 10–20 HU) 10–19 HU = 98.1% PPV for benignity^b^ (3 of 157 malignant) (Note PCC considered benign)	< 2 cm = 94.1% PPV for benignity^b^ (46 of 784 malignant) ≤ **4** **cm** = **93.1% PPV for benignity**^**b**^ (82 of 1190 malignant) 2–4 cm = 91.1% PPV for benignity^b^ (36 of 406 malignant) (Note PCC considered benign)	**None** (but < 20 HU alone = 99.4% PPV for benignity^b^)
Bancos et al [76]	At most, 83.6%^a^	**≤ 20 HU** = **99.8% PPV for benignity**^**b**^ (1 ACC + 1 OM in 1162 < 20 HU, both 10–20 HU) 10–20 HU = 99.1% PPV for benignity^b^ (Note PCC considered benign)	< 2 cm = 99.0% PPV for benignity^b^ (6 of 576 malignant—all OM) < **4** **cm** = **98.6% PPV for benignity**^**b**^ (21 of 1529 malignant) 2 to < 4 cm = 98.4% PPV for benignity^b^ (Note PCC considered benign)	**< 4** **cm and** ≤ **20 HU** = **100% PPV for non-ACC**^**b**^ (possibly also 100% PPV for benignity, as 1 x OM 10–20 HU, but size not stated)
Hong et al [78]	At most 95.5%^b^	**< 19.9 HU** = **100% PPV for benignity**^**b**^ (1 ACC = 19.9 HU, ≥ 4 cm) (Note after excluding PCC, myelolipomas, cysts and other benign)	< 3.5 cm = 100% PPV for benignity^b^ < **4** **cm** = **99.9% PPV for benignity**^**b**^ (1 neuroblastoma 3.5 cm, 38.2 HU; 1 ACC 4.0 cm) (Note after excluding PCC, myelolipomas, cysts and other benign)	**< 20 HU and** < **4** **cm** = **100% PPV for benignity**^**b**^
Kahramangil et al [37]	Possibly all	**≤ 20 HU** = **99.8% PPV for non-ACC**^**b**^ 10–20 HU = 99.5% PPV for non-ACC^b^ (Note no figures for OM)	**< 4** **cm** = **99.9% PPV for non-ACC**^**b**^ 4–6 cm = 97.6% PPV for non-ACC^b^ > 6 cm = 80.5% PPV for non-ACC^b^ (Note no figures for OM)	**None** (but < 4 cm alone = 99.9% PPV for non-ACC^**b**^) (and ≤ 20 HU alone = 99.8% PPV for non-ACC^**b**^)

Bold typeface indicates PPVs closest to 20 HU and 4 cm threshold criteria

Studies ordered by increasing estimated rates of true AIs

*AI* adrenal incidentaloma, *ACC* adrenocortical carcinoma, *PCC* phaeochromocytoma, *HU* Hounsfield unit, *OM* other malignancy, *PPV* positive predictive value

^a^ Provided data

^b^ Calculated from data

^c^ ‘Adenoma’ was defined by size stability ≥ 1 year

First, there are data variances that likely arise from differing statistical reporting—some providing sensitivities of > 20 HU for all malignancy, or only ACC; and conversely, some reporting specificities of < 20 HU for benignity, or solely adenomas. However, where provided or calculable, we report the positive predictive value (PPV) for benignity, given it factors in disease prevalence.

Second, the ESE analysis presumed each study comprised incidentaloma populations; however, upon interrogation, the true AI proportions varied greatly (Table 1). In Ebbehoj et al, 18.4% were identified non-incidentally (e.g., cancer/hormonal workup) [75]; in the EURINE-ACT study, 16.4% had clinically suspected steroid excess or tumours [76]; and in Schloetelburg et al, 31% had extra-adrenal malignancy or suspected adrenal disease [67]. In Marty et al, > 50% either had adrenal biopsy or surgery, highly atypical for an incidentaloma population [77]. Accordingly, in these four studies, the non-PCC malignancy rates reached up to 15.1%.

Nonetheless, despite their heavily surgically enriched population, Marty et al found that < 20 HU maintained a 96.2% PPV for adenomas, improving to 98.6% when combined with a 4 cm cutoff (Table 2) [77]. Schloetelburg et al and Ebbehoj et al had PPVs for benignity for 20 HU of 99.0% and 99.4%, respectively [67, 75]. Even more convincingly, the EURINE-ACT study reported 99.8% PPV for 20 HU, and independently, 98.6% PPV for size < 4 cm [76]. When combined, 20 HU and 4 cm excluded 100% of ACC, and there was only one non-ACC malignancy < 20 HU, with size not stated.

Hong et al had a more appropriate 1.7% malignancy rate, though with 30.5% functional lesions (benign + ACC), including 4.5% with overt Cushing syndrome, and therefore not incidental [78]. Nonetheless, a 19.9 HU threshold was 100% sensitive for malignancy, with a single ACC of that attenuation in 1149 patients, but notably > 4 cm, giving 100% PPV for benignity for 20 HU and 4 cm combined.

Most recently, in the study not reviewed in the 2023 ESE Guidelines, Kahramangil et al found a malignancy rate of 2.2% in 2219 AIs. The ACC risk was only 0.16% for < 20 HU, and independently 0.1% for < 4 cm, and we theorise these combined parameters would approach, if not reach 100% PPV for benignity [37]. Additionally, in a study of exclusively large (> 4 cm) tumours, comprising only 67% true AIs, a 20 HU cutoff still had 98.5% NPV for malignancy [79].

Accordingly, we propose that in true AIs, combined 20 HU and 4 cm cut-offs exclude malignancy, and replace the established 10 HU threshold. We also highlight that even in those > 20 HU but < 4 cm, or > 4 cm but < 20 HU, malignancy rates are also extremely low.

## Where should the focus of future adrenal incidentaloma research be?

Whilst we believe there is sufficient data to support our recommendations thus far, we acknowledge other aspects of AI workup presently lack strong evidence. We therefore encourage further research into these areas to assist in refining an algorithmic approach to AI management.

### Further attenuation and size subcategorisation

Further stratification of subgroups within and beyond the stated 20 HU and 4 cm thresholds (e.g., combinations of 10–20 HU, 20–40 HU; 1–2 cm, 2–4 cm, 4–6 cm, > 6 cm) may enable reduced imaging for additional benign lesions. Some evidence suggests a more cautious approach may be appropriate for all lesions > 40 HU on ncCT [40, 49, 54] given the relative infrequency of adenomas in this range; however, this data was obtained primarily in the context of PCC determination. Additionally, some studies indicate lesions > 6 cm have a significantly increased risk of malignancy, independent of attenuation [36, 37, 79, 80]. Conversely, there is early data that AIs below a size threshold of 1.5 cm may not require cortisol testing; however, larger validation studies are required [81].

### Post-contrast CT detected adrenal incidentalomas

For AIs first detected on post-contrast CT with indeterminate imaging features, ncCT may be performed. However, given the established low ~0.1% risk of malignancy of true AIs < 4 cm of any attenuation [37, 78], it may be possible that ncCT could be delayed 6–12 months in this subgroup to simultaneously allow growth assessment. Additionally, further research may identify subgroups where ncCT may not be required at all.

### Stability and growth

Current guidelines indicate a variable range of 6–12-month stability as evidence of benignity [2, 3, 5], and there is currently only limited data assessing adenoma versus ACC growth rates [82, 83]. Further research into both these areas is therefore warranted.

### Spectral CT attenuation

Historic studies have been based on conventional 120 kVp imaging data; however, attenuation values derived from spectral CT, including virtual noncontrast measurements, may differ [84–87]. With the expected proliferation of photon-counting CT, it will be important to establish how spectral CT attenuation measurements can be used in AL assessment.

### Heterogeneous lesions and morphology

A recent study indicated that attenuation measurement in heterogeneous lesions has poor diagnostic performance [88]. Furthermore, heterogeneity and other morphologic features in some studies have shown high specificity but poor sensitivity for indicating malignancy [89–91]. Further investigation into the management of heterogeneous nodules is therefore warranted, noting recent disappointing results also for MRI in differentiating adenomas and metastases by heterogeneity and T2-weighted signal [59–62].

### Chemical-shift MRI

Chemical-shift (CS) MRI is long-established as an alternative method for detecting microscopic fat, largely assessed qualitatively, though with the most cited quantitative cutoff being a signal intensity index [(in-phase signal − opposed-phase signal)/in-phase signal] ≥ 16.5% [10, 92, 93]. The utility of CS MRI, however, not unexpectedly declines with increasing lesion CT attenuation, particularly ≥ 40 HU [94–97]. Given the availability and cost considerations of MRI, feasibility and efficacy studies into its role in lesions 20–40 HU are therefore suggested, as well as head-to-head comparisons to spectral-CT fat quantification as an alternative CT-based modality [98, 99].

### PET-CT

Data demonstrating higher specificity of 18F-FDG PET-CT than CT attenuation for malignancy has been largely based on cancer populations [100–102] and therefore not applicable to AIs. Furthermore, functional (especially cortisol-secreting) adenomas have been associated with higher FDG uptake [103], as well as those of higher attenuation, reducing specificity in the group that it would be of most potential gain [104]. Nonetheless, there may be a future role of PET-CT, with research into other tracer candidates such as 11C-metomidate, which has specific adrenal cortical uptake [105–107].

### Artificial intelligence

There are multiple preliminary studies describing the use of artificial intelligence in adrenal incidentalomas, primarily in the areas of lesion detection, segmentation and characterisation, with the latter utilising radiomics and machine learning or specifically deep learning; however, most studies are of small populations, and with current limited clinical usage [108]. Larger studies are therefore recommended, particularly those that integrate clinical information, including endocrine laboratory results, with the imaging findings.

### Non-incidental adrenal lesions

We reiterate that our recommendation to cease the use of AWCT applies only to the true AI patient population. Outside of this, further delineation of any remaining role of AWCT is recommended, acknowledging that for those with extra-adrenal malignancy, this may depend on the nature of the primary tumour. It is noted that in the 2023 ESE Guidelines, the diagnostic algorithm for ALs in this clinical context includes biopsy, FDG PET-CT and resection, with no current reference to AWCT [3]. It is also unclear if there is any role for AWCT in patients with functional lesions, given that these are more likely to be managed surgically. Nonetheless, where biopsy and PET-CT are unavailable, it is possible AWCT may still have a contributory role in non-incidental adrenal lesions.

## Summarised recommendations for adrenal incidentaloma management

Based on the presented evidence, and with acknowledgement of areas requiring further research, the following AI management principles are provided:

### Definition of adrenal incidentalomas

A robust definition of an AI inherently delineates a population with an extremely low prevalence of malignancy. To reiterate, we define AIs as ≥ 1 cm lesions in patients without current or prior extra-adrenal malignancy, or clinically suspected adrenal disease. AIs with overtly benign imaging features, such as myelolipomas or cysts, or those with long-term stability (for now, defined as 6–12 months) do not require further evaluation.

### Recommendation for hormonal correlation

Radiology reports should include a recommendation to perform a hormonal evaluation for all remaining AIs, which will identify nearly all PCC, as well as most ACC. Additionally, endocrine correlation identifies subclinically functional adenomas (including MACS), where clinical management may take precedence. Recommending additional endocrinologist referral may depend on local preferences, as in some regions, to manage cost and access issues, endocrine testing can be performed by primary care physicians, with endocrinologist referral limited to those with abnormal results.

### Elimination of adrenal washout CT

In the defined AI population, and with application of endocrine assessment, current evidence indicates no role for AWCT.

### Categorisation on noncontrast CT

**Fig. 1:**
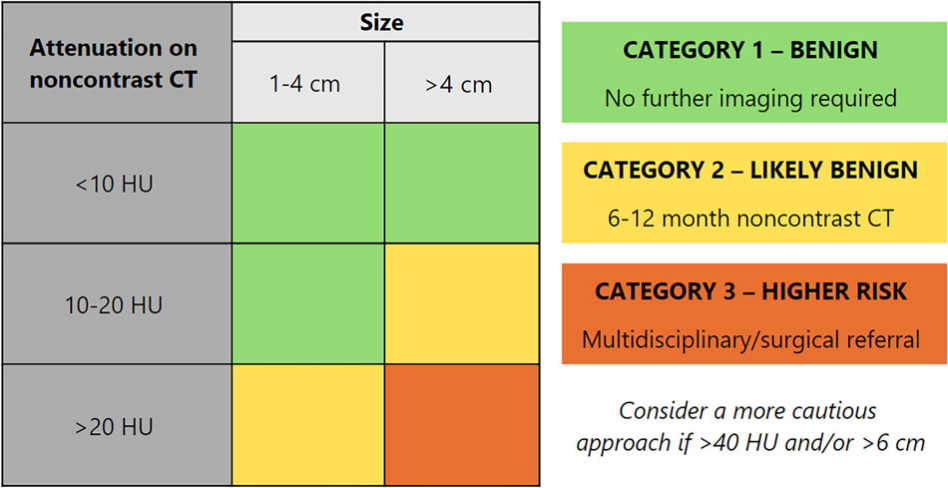
Management of adrenal incidentalomas on noncontrast CT^a^. ^a^ Table only to be used in conjunction with summarised recommendations. HU, Hounsfield unit

On ncCT (Fig. 1), homogeneous AIs ≤ 20 HU and ≤ 4 cm, or as per existing guidelines, < 10 HU and any size, are considered benign (Category-1), with no imaging follow-up required. AIs which are > 20 HU and 1–4 cm, OR 10–20 HU but > 4 cm, are highly likely benign (Category-2), and therefore 6–12 month ncCT is currently suggested as supported by most AI guidelines, purely to identify growth or stability.

Those > 20 HU AND > 4 cm are considered higher risk (Category-3), with multidisciplinary meeting or surgical referral recommended, albeit acknowledging that most will still be of benign aetiology. As discussed prior, a more cautious approach is advised if AIs are > 40 HU and/or > 6 cm.

## Conclusion

Whilst the 10 HU threshold and AWCT have been ingrained in the radiology mindset over the last 2–3 decades, we believe it is now a timely end of an era for both these tenets of AI imaging. First, there is now sufficient evidence that a 20 HU threshold in AIs < 4 cm can safely replace the prior 10 HU limit. Second, due to its inherent inaccuracy and the extremely low incidence of malignancy in true AIs, AWCT has no role in the evaluation of ALs in patients without prior/current malignancy or suspected adrenal disease. Instead, AIs can be managed with ncCT, in parallel with endocrine testing, which complementarily improves detection of benign (and rarely malignant) hormonally active lesions.

We believe these measures will be of significant benefit to patients, radiology departments and healthcare systems in reducing the volume of unnecessary imaging for adrenal lesions. We also encourage further research to identify additional lower-risk subgroups where imaging follow-up or clinical escalation may be unnecessary.

## Supplementary information

ELECTRONIC SUPPLEMENTARY MATERIAL

## Abbreviations

ACC: Adrenocortical carcinoma
AI: Adrenal incidentaloma
AL: Adrenal lesion
APW: Absolute percentage washout
AWCT: Adrenal washout CT
CS: Chemical shift
ENSAT: European Network for the Study of Adrenal Tumors
ESE: European Society of Endocrinologists
HU: Hounsfield units
LPA: Lipid-poor adenoma
LRA: Lipid-rich adenoma
MACS: Mild autonomous cortisol secretion
ncCT: Noncontrast CT
NPV: Negative predictive value
PCC: Phaeochromocytoma
PPV: Positive predictive value
PV: Portal venous
RPW: Relative percentage washout
