# Supplementary material for: Washed up: the end of an era for adrenal incidentaloma CT
Source: Insights Imaging. 2025 Jun 27;16:136. doi: 10.1186/s13244-025-02015-4 (PMC12204974; doi:10.1186/s13244-025-02015-4)
Supplement: Supplementary file 1 — ELECTRONIC SUPPLEMENTARY MATERIAL [file 13244_2025_2015_MOESM1_ESM.docx]

**Supplementary Table S1: Recommended endocrine testing^a^**

| **Applicable Population** | **Recommended Investigation** | **Disease of Interest** |
| --- | --- | --- |
| All adrenal incidentalomas | careful clinical examination for signs & symptoms of adrenal excess | any potential adrenal-related hormonal excess |
| All adrenal incidentalomas | 1mg overnight dexamethasone suppression | cortisol secretion (overt Cushing syndrome or mild autonomous cortisol secretion) |
| All adrenal incidentalomas >10HU  (on non-contrast CT) | plasma or urinary metanephrines | phaeochromocytoma |
| Unexplained hypertension or hypokalaemia | aldosterone/renin ratios | primary hyperaldosteronism |
| Clinical, biochemical or imaging features suggesting adrenocortical carcinoma | sex hormones & steroid precursors | adrenocortical carcinoma |

^a^Derived from Fassnacht et al 2023 [3]

Abbreviations: *HU*, Hounsfield unit
